# A Model Perspective Explanation of the Long-Term Sustainability of a Fully Human BCMA-Targeting CAR (CT103A) T-Cell Immunotherapy

**DOI:** 10.3389/fphar.2022.803693

**Published:** 2022-02-02

**Authors:** Wei Mu, Xiaolu Long, Haodong Cai, Caixia Chen, Guang Hu, Yaoyao Lou, Shugang Xing, Di Wang, Jue Wang, Min Xiao, Kun Wang, Zhongyi Sun, Chunrui Li, Jianfeng Zhou, Liting Chen

**Affiliations:** ^1^ Department of Hematology, Tongji Medical College, Tongji Hospital, Huazhong University of Science and Technology, Wuhan, China; ^2^ Immunotherapy Research Center for Hematologic Diseases of Hubei Province, Wuhan, China; ^3^ Nanjing IASO Biotherapeutics Ltd., Nanjing, China; ^4^ Department of Hematology, School of Medicine, Sir Run Run Shaw Hospital, Zhejiang University, Hangzhou, China; ^5^ Shanghai Qiangshi Information Technology Co., Ltd., Shanghai, China

**Keywords:** populational pharmacokinetics, CAR (chimeric antigen receptor) T cells, immunotharapy, sustainability, modeling

## Abstract

Different from canonical drugs, CAR T-cells are “living drugs”, which derived from patient’s own blood. Studies of the pharmacokinetics of CAR T-cells could improve our understanding of their efficacy, safety, optimal dosage, and other characterizes. We previously reported a phase I study of a novel fully human BCMA-targeting CAR (CT103A) in 18 patients with relapsed/refractory multiple myeloma. CT103A exhibited extraordinary persistence with low anti-drug antibody positivity. To figure out the pharmacokinetic characterizes and investigate the potential reason of CT103A’s long-term persistence, we established a population pharmacokinetic (PopPK) model of CT103A based on 18 patients cohort by applying nonlinear mixed-effects modeling and analyzed the CAR T-cell clonal evolution. The results suggested that extramedullary spreading was found to impair C_max_ and was therefore added as a covariate to the modified model. The model revealed tocilizumab and corticosteroids showed no impact on the CT103A expansion rate. No dominant clone existed in patients with persistently high peripheral CT103A by CAR integration sites analysis. Finally, patients with lower contraction rate constants and higher C_max_ as well as memory CT103A fraction could achieve better clinical responses. Taken together, this study developed a PopPK model of a fully human anti-BCMA CAR T-cell therapy, and summarized its model characteristics. We suggested that the long-term persistence of CT103A was attributed to the memory CAR T-cell fraction but not the clonal evolution. This study will improve people’s understanding of pharmacokinetics and PopPK of CAR T-cell immunotherapy.

## Introduction

Pharmacokinetics is a branch of pharmacology dedicated to describing the fate of drugs administered to patients, including the extent and rate of their absorption, distribution, metabolism and excretion (Whiting et al., 1986). As individual pharmacokinetics may vary over time and most variability in the pharmacokinetic parameters within individuals is not predictable, it is important to study pharmacokinetics at the population level (Currie, 2018). As a “living drug” derived from the patient’s own blood, CAR T-cells do not follow the typical pharmacokinetic models of canonical drugs (Grupp et al., 2013; Stein et al., 2019). Thus, studies of the pharmacokinetics of CAR T-cells could improve our understanding of their efficacy, safety and optimal dosage.

Several studies have analyzed the pharmacokinetics and population pharmacokinetics (PopPK) of tisagenlecleucel, an FDA-approved anti-CD19 CAR T-cell product (Mueller et al., 2017; Mueller et al., 2018; Stein et al., 2019; Awasthi et al., 2020). A PopPK model was also reported for the kinetics of tisagenlecleucel based on data collected from two phase II studies (ELIANA and ENSIGN) (Stein et al., 2019). However, different CAR T-cell products vary in expansion rate, distribution, persistence and other pharmacokinetic characterizations, especially CAR T-cell products targeting different antigens. Therefore, it is still worth establishing PopPK models of CAR T-cells targeting antigens other than CD19. On the other hand, the tisagenlecleucel model was focused on pediatric and young adult patients with relapsed/refractory B cell acute lymphoblastic leukemia. We were concerned about PopPK in patients with different baseline characteristics and other hematological malignancies.

We previously reported a phase I study of a novel fully human BCMA-targeting CAR (CT103A) in 18 patients with relapsed/refractory multiple myeloma (http://www.chictr.org.cn, Number, ChiCTR1800018137) (Wang et al., 2021). The median CT103A transgene persistence was 307.5 days. At 1 year, only one patient was positive for the anti-drug antibody (ADA). In this study, we were also aimed to figure out the potential reason of CT103A’s long-term persistence.

We established a PopPK model of CT103A based on this patient cohort (*n* = 18), analyzed covariates that impact the parameters and evaluated optimal dosage. Moreover, effects of therapies for treating cytokine release syndrome (CRS) (tocilizumab and corticosteroids) on CAR-T expansion rate were calculated. Finally, as some patients exhibited long-lasting high peripheral CT103A, we monitored the CAR integration sites to reveal the clonal population structure. Our results suggested that the long-term persistence of CT103A may not due to clonal evolution but the memory CAR T-cell fraction. This study will improve people’s understanding of pharmacokinetics and PopPK of CAR T-cell immunotherapy.

## Materials and Methods

### Absolute Quantification of Peripheral CT103A by Droplet Digital Polymerase Chain Reaction

CAR gene specific Minor Groove Binder (MGB) conjugated DNA probe was designed by Primer Express 3.0. Absolute copy numbers of CAR gene in the peripheral blood of patients were quantified using droplet digital polymerase chain reaction (ddPCR) at multiple time points after infusion of CAR-T cells. The ddPCR results were analyzed using QuantaSoft software version 1.7.4 (Bio-Rad), according to the manufacturer’s recommenda-tions. The droplet reader software results were represented as copies/μg DNA for CAR-transgene.

### Model- Based Analysis

The base structural model is adapted from a previously published tisagenlecleucel model. The structural model captures the exponential expansion of CT103A with rate constant *ρ* up to time T max, followed by biexponential contraction after T_max_ with rate constants *α* and *β*. The base model has the following six fixed parameters: C_max_, T_max_, fold x, F B, *α*, and *β*.

### Covariate Analysis

Covariate analysis was conducted after the selection of the base model. Scatter plots of parameter estimates from the selected base model and potential covariates (including extramedullary spreading) were plotted to explore covariate-parameter relationships. The Stepwise Covariate Model (SCM) module was then used for covariate screening and identification. Covariate relationships as power function were assessed for continuous covariates, and a stepwise approach was used to evaluate covariate effects. The statistical criteria for a covariate to be incorporated in the model were a decrease of >3.84 in the OFV (*p* = 0.05, χ2 distribution with one degree of freedom) in the forward step and/ an increase of <6.63 in the OFV (*p* = 0.01, χ2 distribution with one degree of freedom) in the backward step.

### Exposure-Response Analysis

The probability of response versus dose in CT103A treated patients was plotted, with probabilities calculated across sets of patients binned by dosage group. The binary efficacy endpoints were analyzed by logistic regression models if an exposure-response trend is observed. Exposure-response analysis was performed with R 3.6.1.

### Calculation of Effector and Memory CT103A

Effector and memory CT103A represent initial fast declining and subsequent persistent CT103A, respectively. Their values were calculated using the Eqs below:

$$\frac{dY_{\alpha }}{\mathrm{dtime}}=\{\begin{array}{l} \frac{\ln\left(fold_{x}\right)}{T_{\max}}\left(\mathrm{time}=\text{ 0}\right)\\ \frac{\ln\left(fold_{x}\right)}{T_{\max}}\times Y_{\alpha }\left(0\;\text{<}\;\mathrm{time}\leq T_{\max}\right)\\ -\alpha \times Y_{\alpha }\left(\mathrm{time}\text{>}\;T_{\max}\right) \end{array}$$

$$\frac{dY_{\beta }}{\mathrm{dtime}}=\{\begin{array}{l} 0\;\;\;\;\;\;\;\;\;\;\;\;\;\;\;\;\;\;\;\;\;\;\;\;\;\;\;\;\;\;\;\;\;\;\;\;\;\;\;\;\;\;\;\;\;\;\;\;\;\;\;\;\;\;\;\;\;\;\;\;\left(\mathrm{time}\leq T_{\max}\right)\\ fb\times \left(\alpha -\beta \right)\times Y_{\alpha }-\beta \times Y_{\beta }\;\;\;\;\;\;\;\;\;\;\left(\mathrm{time}\text{ > }T_{\max}\right) \end{array}$$

## Results

### Patients Characteristics

The baseline characteristics of the included patients are listed in Table 1. Their median age was 53.5 years (range, 38–66), with 38.9% aged ≥ 60 years. Ten patients are male and 8 patients are female. Five patients had extramedullary multiple myeloma (EMM) (27.8%). The definition of EMM is the presence of soft tissue masses in extraosseous locations resulting from hematogenous spread, which are not contiguous to the involved bone (Bhutani et al., 2020; Wang et al., 2021). Six patients (33.3%) had previously undergone auto-HSCT while four patients (22.2%) had received prior murine anti-BCMA CAR T-cell treatment (http://www.chictr.org.cn, Number, ChiCTR-OPC-16009113). CT103A was administered at 1, 3, and 6 × 10^6^ CAR-positive T cells/kg. In total, 17 out of 18 (94.4%) patients experienced CRS, of which 12 (12/17, 70.6%) were grades 1 and 2.4 (4/17, 23.5%) and 1 (1/17, 5.9%) patients experienced grade 3 and grade 4 CRS separately. Eleven patients (61.1%) underwent tocilizumab and twelve patients (66.7%) underwent corticosteroid therapy for treatment of CRS.

**TABLE 1:** Patient and disease characteristics at baseline.

Characteristic	
**Age**, years	
Mean	54
Median	53.5
Range	38–66
**Age group**	
Younger than 60 y	11 (61.1%)
At least 60 y	7 (38.9%)
**Gender**	
Male	10 (55.6%)
Female	8 (44.4%)
**Nation**	
Han	18 (100%)
**Dose of CAR T-cells**	
1.0*10^6 cells/kg	9 (50.0%)
3.0*10^6 cells/kg	6 (33.3%)
6.0*10^6 cells/kg	3 (16.7%)
**Number of lines of previous therapy**	
3	4 (22.2%)
4	9 (50.0%)
5	4 (22.2%)
6	1 (5.6%)
**Previous auto-HSCT**	
Yes	6 (33.3%)
No	12 (66.7%)
**Previous CAR T**	
Yes	4 (22.2%)
No	14 (77.8%)
**Extramedullary lesions**	
Yes	5 (27.8%)
No	13 (72.2%)
**Treated with tocilizumab during therapy**	
Yes	11 (61.1%)
No	7 (38.9%)
**Treated with corticosteroids during therapy**	
Yes	12 (66.7%)
No	6 (33.3%)

### PopPK Base Model of CT103A

**FIGURE 1:**
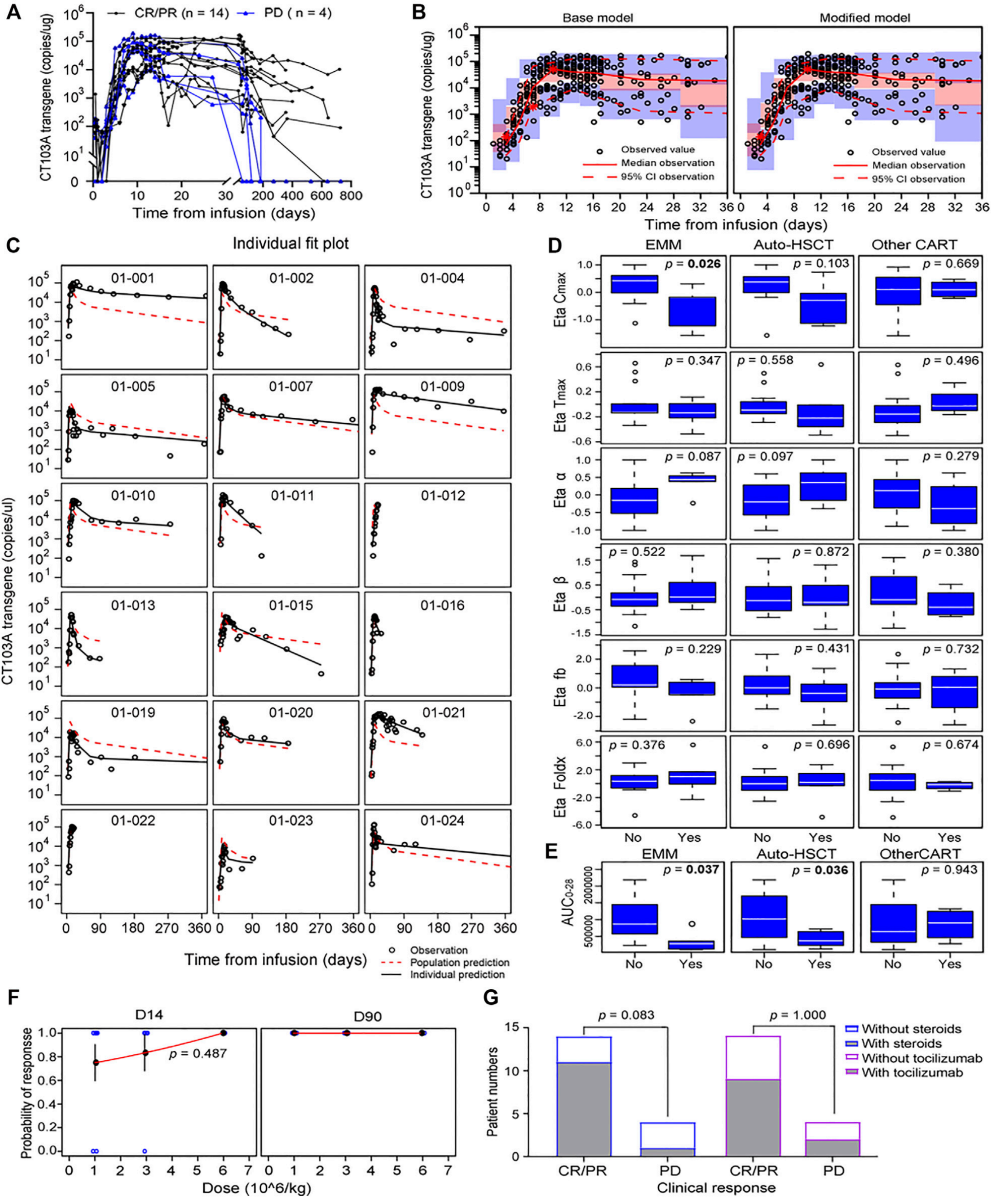
Model-based pharmacokinetic analysis of CT103A. **(A)** Pharmacokinetics of peripheral transgene by 1 year clinical response in the CT103A clinical study. **(B)** Visual predictive checks of the base model (left) and modified model (right) simulation compared with the observed data. Hollow circles represent observed values, the solid lines and the dashed lines represent the median and the 95% CI of observation, respectively. The middle red shadow areas represent the 90% confidence intervals of median for the results of 1,000 times simulation and the blue shadow areas represent the 90% confidence intervals of the 2.5th and 97.5th percentiles of the results of 1,000 times simulation. **(C)** Impacts of extramedullary spreading and prior therapies (prior other CAR T-cell therapy and auto-HSCT) on Bayesian post hoc random effects estimates. EMM, extramedullary multiple myeloma. **(D)** Impacts of extramedullary spreading and prior therapies (prior other CAR T-cell therapy and auto-HSCT) on AUC_0-28_. **(E)** Individual fit plots for CT103A transgene copy number over time. **(F)** Probabilities of responses versus doses at day 14 and day 90 after infusion were plotted. Probabilities at day 14 were fitted to a logistic regression with standard error bars showed. Blue hollow circles represent observed data; Red lines represent estimated data. **(G)** One-year clinical responses with/without steroids (left) and with/without tocilizumab (right).

In this study, the cellular kinetics of CT103A were determined by both transgene measurement by droplet digital PCR (ddPCR) and the percentage of CD3^+^ cells expressing the CAR receptor in lymphocyte by flow cytometry (Lou et al., 2020). As there was a significant linear correlation between flow cytometry- and ddPCR-based measurements (*p* < 0.0001, Supplementary Figure S1), only the ddPCR data was used in the following pharmacokinetic analysis. A total of 469 measurements, 41 of which were below the limit of quantitation (20 copies/μg gDNA), were used to develop the base model. The cellular kinetics of the CT103A transgene in 18 patients are shown in Figure 1A.

The CT103A PopPK structure base model was adapted from the tisagenlecleucel model and developed by applying a nonlinear mixed-effects model (NONMEM) (Currie, 2018; Stein et al., 2019). Following infusion, the copy number of CT103A declined in the first few days and then exhibited exponential growth at rate *ρ*. After reaching the maximal concentration C_max_ at time T_max_, the kinetics of CT103A expansion were followed by a biexponential contraction including an initial rapid contraction (with constant rate *α*) and subsequent persistent contraction (with constant rate *β*) (De Boer and Perelson, 2013). The initial contraction occurred at a rapid rate and was thought to correspond to the quick decline of effective CAR-T cells population. The second phase of CT103A decline occurred more gradually over time, which was thought to be caused by the persistence of memory CAR T-cells population. Fb was the fraction of CT103A with contraction rate *β* at peak expansion (T_max_). The formulation of CT103A PopPK base model is given in Eq. 1 as follows:

$$\begin{array}{l} \rho =\frac{ln\left(fold_{x}\right)}{T_{\max}}\\ f\left(t\right)=\{\begin{array}{l} \frac{C_{max}}{fold_{x}}\times e^{\rho \times time}\;\;\;\;\;\;\;\;\;\;\;\;\;\;\;\;\;\;\;\;\;\;\;\;\;\;\;\;\;\;\;\;\;\;\;\;\;\;\left(\mathrm{time}\;\text{<}\;T_{\max}\right)\\ C_{max}\times \left(1-fb\right)\times e^{-\alpha \times \left(time-T_{max}\right)}+C_{max}\times fb\times e^{-\beta \times \left(time-T_{max}\right)}\;\;\;\;\;\;\;\left(\mathrm{time}\geq T_{\max}\right) \end{array} \end{array}$$ (1)

Model evaluation was assessed using a prediction-corrected visual predictive check (pcVPC) and individual fits (Figure 1B left panel and Figure 1C). Additional diagnostics are included in the supplementary material (Supplementary Figure S2). The fixed effects and random effects of the main parameters, namely, C_max_, T_max_, foldx, fb, *α* and *β*, were calculated and are listed in Supplementary Table S1. The initial doubling time (ln 2/*ρ*) and the half-lives for the initial (ln 2/*α*) and terminal (ln 2/*β*) declines were 1.78, 6.04 and 149.39 days, respectively.

### Covariates Evaluation and Model Modification

**FIGURE 2:**
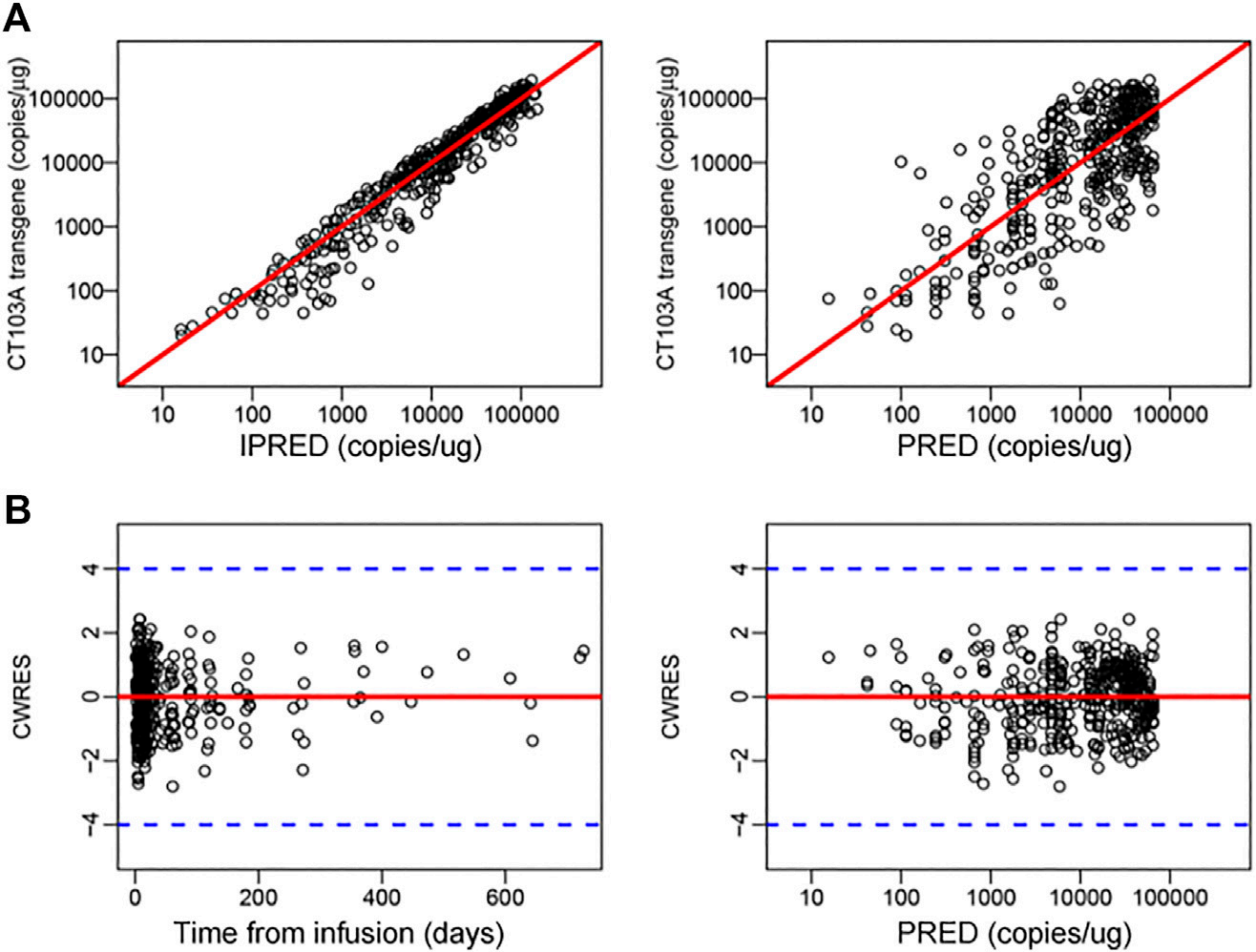
Diagnostics for modified m41odel. **(A)** Individual predicted concentration versus observed concentration (left). Population predicted concentration versus observed concentration (right). The red lines represent regression lines. **(B)** Conditional weighted residuals versus time (left) and population predicted concentration (right). The red lines and blue dash lines represent the position where conditional weighted residual equal 0 and ± 4, respectively.

To explore the influence of extrinsic and intrinsic factors on the CT103A PopPK base model, we then conducted covariate evaluation for patient demographics (age, gender, and weight), prior therapies (prior murine anti-BCMA CAR T-cell treatment (http://www.chictr.org.cn, Number, ChiCTR-OPC-16009113) and autologous hematopoietic stem cell transplantation (auto-HSCT)), extramedullary spreading (Bhutani et al., 2020; Sevcikova et al., 2019), dose and CRS grade (Figure 1D; Supplementary Figure S3; Supplementary Table S2). The results revealed that among these extrinsic and intrinsic factors, only extramedullary spreading statistically significantly impacted C_max_. Patients who had extramedullary myeloma achieved a significantly lower C_max_ than patients without extramedullary spreading (*p* = 0.0258). This result was consistent with our previously reported Kaplan-Meier analysis of the same patient cohort, which indicated that extramedullary spreading was associated with a shortened PFS (*p* = 0.015) (Wang et al., 2021). Furthermore, we also explored the impacts of all the factors on the area under the curve over the first 28 days after infusion (AUC_0-28_). Our results showed that patients with prior auto-HSCT or extramedullary spreading had a significantly lower AUC_0-28_ (*p* = 0.0357 and 0.0372, respectively) (Figure 1E). After including extramedullary spreading as a covariate, a modified model was constructed (Table 2), and its diagnostics are shown (Figure 1B right panel and Figure 2). The modified model formulation is shown in Eq. 2 below:

$$\begin{array}{l} C_{\max}^{\prime }=C_{\max}\times \theta_{\mathrm{extramedullary}}\left(\mathrm{extramedullary}==yes\right)\\ \rho =\frac{ln\left(fold_{x}\right)}{T_{\max}}\\ f\left(t\right)=\{\begin{array}{l} \frac{C_{\max}^{\prime }}{fold_{x}}\times e^{\rho \times time}\;\;\;\;\;\;\;\;\;\;\left(\mathrm{time}\text{<}T_{\max}\right)\\ C_{\max}^{\prime }\times \left(1-fb\right)\times e^{-\alpha \times \left(time-T_{max}\right)}+C_{\max}^{\prime }\times fb\times e^{-\beta \times \left(time-T_{max}\right)}\;\;\;\;\;\;\;\;\left(\mathrm{time}\geq T_{\max}\right) \end{array} \end{array}$$ (2)

**TABLE 2:** Modified model PK parameters.

Type	Parameter	Units	Estimate	RSE (%)	Eta shrinkage
Fixed effect	foldx	—	5,460	0.8077	—
Fixed effect	fb	—	0.1172	0.1014	—
Fixed effect	Alpha	1/day	0.1093	0.3528	—
Fixed effect	Beta	1/day	0.006122	0.08901	—
Fixed effect	C_max_	Copy/ug	67,060	17.94	—
Fixed effect	T_max_	Days	8.657	0.1016	—
Cmax covariate effect	Extramedullary spreading	—	0.468	17.94	—
Random effect	foldx	—	215.6	94.42	1.00E-10
Random effect	fb	—	154	64.87	9.572
Random effect	Alpha	—	73.49	5.49	23.69
Random effect	Beta	—	107.3	79.09	23.61
Random effect	C_max_	—	59.49	1.585	2.192
Random effect	T_max_	—	29.01	1.59	1.00E-10
Residual error	Sigma proportional	—	47.01	0.8616	—
Residual error	Sigma additive	—	0.319	0.07648	—

C_max_, maximal peripheral CT103A transgene copy number; T_max_, time to maximal expansion; fb, the fraction of persistent CT103A (with contraction rate *β*) at peak expansion (T_max_); foldx, fold expansion from baseline; RSE, relative standard error of the parameter; Eta shrinkage, shrinkage of empirical Bayes estimates of the parameter, Eta shrinkage for each parameter is calculated by the formula (1−var(η))/ω 2.

### Dose Optimization

To aid in the optimization of dosing strategies, we constructed a logistic regression model for the dose-response curve (Figure 1F). No increasing probability of response at day 14 for higher dose was found (*p* = 0.487). Moreover, the probability of response at day 90 was 100% for all the doses. In addition, the incidence of ≥ grade 3 CRS was significantly higher in 6 × 10^6^ CAR T-cells/kg dose group than in the other two dose groups, as previously reported. Thus, for both efficiency and safety reasons, 1 × 10^6^ CAR T-cells/kg is the recommended dose for CT103A. It is worth noting that these dose optimization results are only valid for this fully human CAR T-cell for BCMA.

### Impact of Tocilizumab/Corticosteroids on Expansion and Long-Term Efficacy of CT103A

Tocilizumab and corticosteroids are used frequently to treat CRS, an on-target adverse effect of CAR T-cell therapy (Lee et al., 2014; Neelapu et al., 2018). However, whether tocilizumab or corticosteroids influence the expansion of CAR T-cells is controversial (Davila et al., 2014; Shank et al., 2017; Awasthi et al., 2020; Liu et al., 2020). Moreover, the influence of tocilizumab and corticosteroids on the expansion of CAR T-cells targeting BCMA in multiple myeloma patients has never been reported. In this study, dexamethasone (10 mg/dose, range 5 ∼ 20 mg/dose) or methylprednisolone (40 mg/dose, range 10 ∼ 200 mg/dose) were administered intravenously for CRS management in 12 patients. The median cumulative dexamethasone—equivalent dose was 40 mg (range 10 ∼ 198.75 mg) and the median duration of corticosteroid treatment was 3 days (range 1 ∼ 18 days). Moreover, 11 patients received tocilizumab intravenously at 8 mg/kg of bodyweight (maximum 720 mg in this study) to treat CRS. Herein, we determined the effect of these two comedications on the expansion rate *ρ* of CT103A based on the PopPK model (Eq. 3). T1 and T2 were defined as the first administration times of tocilizumab and corticosteroids, respectively. F1 and F2 were defined as the effects of tocilizumab and corticosteroids on the expansion rate and were calculated by using NONMEM as 0.966 and 0.944, both of which are close to 1 (Supplementary Table S3). Thus, neither tocilizumab nor corticosteroids were found to impact the rate of CT103A expansion. In addition, the long-term efficacy was not affected by tocilizumab or corticosteroid administration (Figure 1G). The remission rates of the steroid group and nonsteroid group were 91.67% (11/12) and 50% (3/6), respectively, while they were 81.82% (9/11) and 71.43% (5/7) in the tocilizumab group and non-tocilizumab group, respectively.

$$\begin{array}{l} \rho =\frac{ln\left(fold_{x}\right)}{T_{\max}}\\ f\left(t\right)=\{\begin{array}{l} \frac{C_{max}}{fold_{x}}\times e^{\rho \times time}\;\;\;\;\;\;\;\;\;\;\;\;\;\;\;\;\;\;\;\;\;\;\;\;\;\;\;\;\;\;\;\;\;\;\;\;\;\;\;\;\;\;\;\;\;\;\;\;\;\;\;\;\;\;\;\;\;\;\;\;\;\;\;\;\;\;\;\;\;\;\;\;\;\;\;\;\;\;\;\left(\mathrm{time}\;\text{<}\;T_{\mathrm{toci}}\right)\\ \frac{C_{max}}{fold_{x}}\times e^{\rho \times T_{toci}}\times e^{F_{1}\times \rho \times \left(time-T_{toci}\right)}\;\;\;\;\;\;\;\;\;\;\;\;\;\;\;\;\;\;\;\;\;\;\;\;\;\;\;\;\;\;\;\;\;\;\;\;\;\;\;\;\;\;\;\;\;\;\left(T_{toci}\leq \mathrm{time}\;\text{<}\;T_{ster}\right)\\ \frac{C_{max}}{fold_{x}}\times e^{\rho \times T_{toci}}\times e^{F_{1}\times \rho \times \left(T_{ster}-T_{toci}\right)}\times e^{F_{1}\times F_{2}\times \rho \times \left(time-T_{ster}\right)}\;\;\;\;\;\;\;\;\;\;\;\;\;\;\;\left(T_{ster}\leq \mathrm{time}\text{ <}\;T_{\max}\right)\\ C_{max}\times \left(1-fb\right)\times e^{-\alpha \times \left(time-T_{max}\right)}+C_{max}\times fb\times e^{-\beta \times \left(time-T_{max}\right)}\;\;\;\;\;\;\;\;\left(\mathrm{time}\geq T_{\max}\right) \end{array} \end{array}$$ (3)

### Clonal Diversity and Effector/Memory Fraction Analysis

**FIGURE 3:**
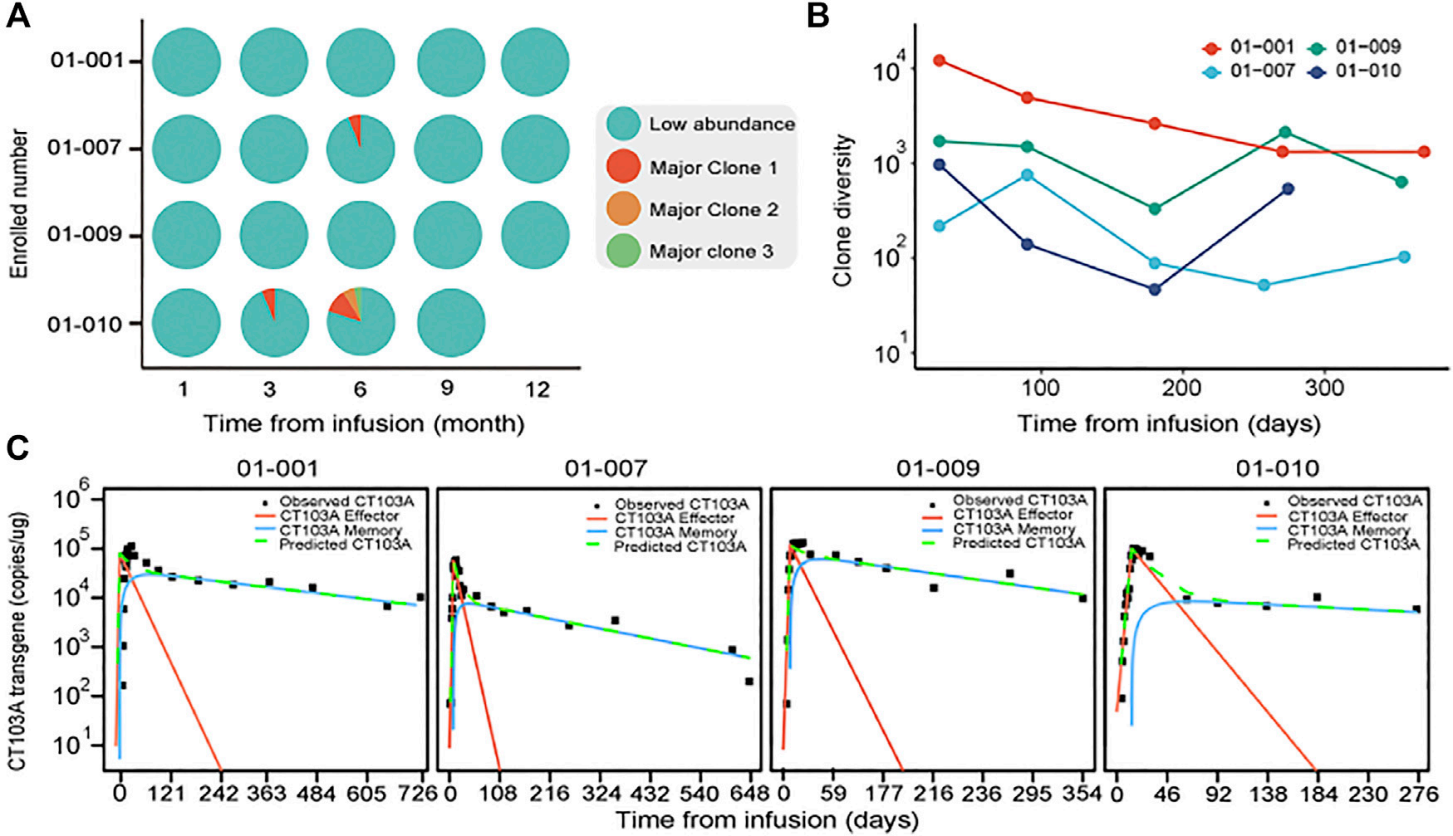
Clonal diversity and effector/memory fraction analysis in patients with long-lasting high level peripheral CT103A. **(A)** The clonal integration sites distributions analysis in patients with consistently high CT103A transgene copy number (>2000 copy/μg DNA) for at least 9 months after infusion. Cyan, indicates collection of low abundant (<3% proportional abundance) clones while red, orange and green indicate the first, second and third major clones in each sample (≥3% proportional abundance) respectively. **(B)** Diversity dynamics of CAR T-cells in recipients with consistently high peripheral transgene copy number. The *x* axis shows the time points sampled; the *y* axis shows the diversity of each clone measured by Simpson’s reciprocal index of diversity. **(C)** Observed CT103A kinetics and predicted kinetics of effector and memory CT103A. Black squares, observed data; green dash lines, model predicted data; red lines, effector CT103A; blue lines, memory CT103A.

As four patients (patients 001, 007, 009, and 010) exhibited long-lasting high level peripheral CT103A (above 2,000 copies/μg gDNA for at least 9 months after infusion), we monitored the clonal origination by analyzing the CAR integration sites, which was described in supplementary methods. It is notable that the clonal diversities in all these subjects didn’t show a salient drop after infusion (Figures 3A,B). This finding suggested that no dominant clone existing in CT103A of these patients. Furthermore, we analyzed the components of CAR-T population of these four patients (Kalos et al., 2011; De Boer and Perelson, 2013; Hardiansyah and Ng, 2019). Their CAR T-cell population was divided into effector CAR T-cells (red line), with a quick activation and extraction, as well as memory CAR T-cells (blue line), and with a slow expansion and long persistence (Figure 3C). All these four patients showed a high fraction of memory CAR T-cells after T_max_.

Finally, we compared the PopPK model constants in patients with different 1-year clinical responses (Supplementary Table S4). The mean values of both the initial and persistent contract constants (*α* and *β*) in the PD group were higher than those in the CR/PR group, which showed that CT103A declined more quickly in the PD group. Moreover, the mean values of fb and C_max_ were lower in the PD group than in the CR/PR group. However, none of these differences was statistically significant, which may be due to the small number of PD patients (*n* = 4).

In summary, by using NONMEM, we established a PopPK model of a fully human anti-BCMA CAR T-cell therapy (CT103A) based on 18 patients from a phase I clinical study. Extramedullary spreading was found to impair C_max_ and was therefore added as a covariate to the final model. Since the higher dose was significantly related to a higher incidence of severe CRS but not with an increasing probability of response for CT103A, the lower dose (1 × 10^6^ CAR T-cells/kg) is recommended. Tocilizumab and corticosteroids showed no impact on the CT103A expansion rate or 1-year clinical responses. No dominant clone existed in patients with persistently high peripheral CT103A. Finally, patients with lower contraction rate constants and higher C_max_ as well as memory CT103A fraction could achieve better clinical responses.

## Discussion

As CAR T-cell therapy has dramatically shifted the landscape of treatment for lymphoid malignancies, there is greater demand for pharmacokinetic research in this field. Till now, very few studies have focused on the PopPK of CAR T-cell therapy. Only an anti-CD19 CAR T, tisagenlecleucel, has been analyzed by mathematically modeling (Stein et al., 2019). In this study, we developed the PopPK model based on 18 patients. Although the patient cohort is small, intensive testing of CT103A transgene numbers were conducted, the PopPK model developed based on this data was diagnosed very well.

Here, we developed a PopPK modeling of a recently published novel fully human BCMA-targeting CAR (CT103A) (Wang et al., 2021). However, the PopPK model and dose optimization are only valid for this CAR-T cell for BCMA. In the previous study, the overall response rate (ORR) of CT103A was 100%, with 72.2% of the patients achieving complete response or stringent complete response (sCR). At 1 year, CAR transgenes were detectable in 77.8% of the patients. The median CAR transgene persistence was 307.5 days. Only one patient was positive for the anti-drug antibody. The good performance of long persistence and low immunogenicity may be partly attributed to the fully human BCMA antibody constructed in CT103A. This long-term sustainability of CT103A may due to the fully human composition without immunogenicity induced by different species. For patients treated with some other anti-BCMA CAR products, the CAR transgene concentration mostly fell below the level of quantification in peripheral blood within 6 months after infusion (Berdeja et al., 2021; Munshi et al., 2021). On the other side, the high memory CAR T-cells fraction made an import role.

In the tisagenlecleucel model previous reported (Stein et al., 2019), no covariates had a statistically significant impact on the cellular kinetic parameters of tisagenlecleucel expansion, thus tisagenlecleucel’s final cellular kinetic model was the same as the base model. However, different from the tisagenlecleucel model, extramedullary spreading statistically significantly impacted C_max_, and has been added as a covariate to the final model. Parameter *θ*_extramedullary_ (extramedullary = yes) was used to modify the base model. Previous studies reported that extramedullary multiple myeloma is associated with high-risk features, such as high LDH levels, high-risk cytogenetics or high-risk gene expression profiles and portends poor prognosis (Usmani et al., 2012; Bhutani et al., 2020).

Impact of tocilizumab and corticosteroids on expansion of CAR T-cells is controversial. Marco L. Davila et al. reported that administration of lymphotoxic high-dose steroids to treat CRS resulted in rapid ablation of anti-CD19 CAR-T cells (Shank et al., 2017). On the other hand, some other studies reported that the steroid group had larger exposure and longer median duration of functional persistence of CAR-T cells compared to non-steroid group (Davila et al., 2014). In addition, a PopPK model-based study showed that tocilizumab and corticosteroids have no impact on tisagenlecleucel’s expansion rate (Stein et al., 2019). In this study, our results suggested that tocilizumab and corticosteroids have no effect on both expansion rate and long-term efficacy of CT103A.

In conclusion, this study developed a PopPK model of a novel fully human BCMA-targeting CAR (CT103A) in 18 patients with relapsed/refractory multiple myeloma. The model constants were calculated. Extramedullary spreading was found to impair C_max_ and was therefore added as a covariate to the modified model. It is suggested that effects of therapies for treating CRS (tocilizumab and corticosteroids) showed no impact on the CT103A expansion rate. We suggested that the long-term persistence of CT103A was attributed to the memory CAR T-cell fraction but not the clonal evolution. Our study will improve people’s understanding of pharmacokinetics and PopPK of CAR T-cell immunotherapy.

## Data Availability

The original contributions presented in the study are included in the article/Supplementary Material, further inquiries can be directed to the corresponding authors.
