# Supplementary material for: A Model Perspective Explanation of the Long-Term Sustainability of a Fully Human BCMA-Targeting CAR (CT103A) T-Cell Immunotherapy
Source: Front Pharmacol. 2022 Feb 2;13:803693. doi: 10.3389/fphar.2022.803693 (PMC8847740; doi:10.3389/fphar.2022.803693)

**Supplemental Materials**

This Supplemental Materials is provided by the authors for additional information about their work.

Supplements to Wei Mu, *et al*. A model perspective explanation of the long-term sustainability of a Fully Human BCMA-Targeting CAR (CT103A) T-Cell Immunotherapy.

**CONTENTS:**

[**SUPPLEMENTAL MATERIAL** 3](#_Toc72401304)

[Visual predictive check 3](#_Toc72401305)

[NONMEM control stream file 3](#_Toc72401306)

[CAR integration sites detection and analysis 5](#_Toc72401309)

[**SUPPLEMENTAL TABLES** 6](#_Toc72401310)

[Table S1: Base model PK parameters 6](#_Toc72401311)

[Table S2: Impact of covariates on the Bayesian posthoc PK parameters from the PopPK base model 7](#_Toc72401312)

[Table S3: F1 / F2 model PK parameters 8](#_Toc72401313)

[Table S4: Summary of modified model individual parameters 9](#_Toc72401314)

[**SUPPLEMENTAL FIGURES** 10](#_Toc72401315)

[Figure S1 Good correlation between ddPCR-based quantification of CT103A transgene copy number and flow cytomatry-based CT103A percentage in lymphocytes. 10](#_Toc72401316)

[Figure S2 Diagnostics for base model. 11](#_Toc72401317)

[Figure S3 Impacts of categorical covariates on Bayesian post hoc random effects estimates and AUC_0-28_. 12](#_Toc72401318)

**SUPPLEMENTAL MATERIAL**

**Visual predictive check**

Visual predictive check (VPC) was used to graphically assess the appropriateness of the compartment model because of the importance of the ability of the model to simulate data similar to the original data. The concentration profiles were simulated 1000 times and compared with observed data to evaluate the predictive performance of the model.

**NONMEM control stream file**

$PROBLEM CT103A

$INPUT C ID TIME DV DOSE GENDER AGE WT CRS Extramedullary Autotransplant otherCART toci ster Ttoci Tster

$DATA input1.csv IGNORE=C

$PRED

TVfoldx =THETA(1)

TVfb=THETA(2)

TValpha=THETA(3)

TVbeta=THETA(4)

foldx =TVfoldx*EXP(ETA(1))

fb=TVfb*EXP(ETA(2))

alpha=TValpha*EXP(ETA(3))

beta=TVbeta*EXP(ETA(4))

CMAX=(THETA(5)*THETA(7)**Extramedullary)*EXP(ETA(5))

TMAX=THETA(6)*EXP(ETA(6))

P=(log(foldx)/TMAX)

IF (TIME.LT.TMAX) THEN

IPRED=(CMAX/foldx)*EXP(P*TIME)

ENDIF

IF(TIME.GE.TMAX) THEN

IPRED=CMAX*(1-fb)*EXP(-alpha*(TIME-TMAX))+CMAX*fb*EXP(-beta*(TIME-TMAX))

ENDIF

Y=IPRED*(1+EPS(1))+EPS(2)

IF (Y.LE.15) THEN

Y=7.5

ENDIF

$THETA (0.001,5460.4) ; foldx

(0.0001,0.117207,1) ; fb

(0.000114,0.109265,100) ; alpha

(0.00001,0.00612234,100) ; beta

(0,67060.5) ; CMAX

(0,8.65658) ; TMAX

(0,0.46805) ; Extramedullary

$OMEGA 4.64825 ; PPV_foldx

2.37135 ; PPV_fb

0.540133 ; PPV_alpha

1.1511 ; PPV_beta

0.353954 ; PPV_Cmax

0.084185 ; PPV_Tmax

$SIGMA 0.220986 ; RUV_CV

0.10174 ; RUV_SD

$ESTIMATION METHOD=COND INTER MAX=9990 NSIG=3 SIGL=9 PRINT=1 NOABORT

$COVARIANCE MATRIX=R

$TABLE C ID TIME DV DOSE GENDER AGE WT CRS Extramedullary Autotransplant OtherCART Toci Ster Alpha Beta Fb Foldx CMAX TMAX IPRED ETA(1) ETA(2) ETA(3) ETA(4) ETA(5) ETA(6) CWRES ONEHEADER NOPRINT FILE=RESmodel01

**CAR integration sites detection and analysis**

Genomic DNA samples from PBMC used for vector integration sites detection were collected regularly after infusion. Integration sites detection was conducted as modified INSPIIRED pipeline. Briefly, genomic DNA was randomly sheared and ligated with linker and worked as template for nested PCR to specifically amplify vector integrated fragments. Sample libraries were constructed with PCR products and sequenced by NovaSeq 6000 (Illumina) after passing quality control. An in-house R script was used to process fastq files to generate final integration sites coordinates. The script contains sequencing reads quality filtering, barcodes and unique molecular identifiers (UMIs) extraction, non-genomic sequences trimming, alignment, PCR duplicates removing, UMI and breaking-point based filtering. Clonal diversity was measured as the reciprocal of Simpson’s Diversity Index.

**SUPPLEMENTAL TABLES**

**Table S1: Base model PK parameters**

| Type | Parameter | Units | Estimate | RSE (%) | Eta Shrinkage |
| --- | --- | --- | --- | --- | --- |
| Fixed effect | foldx | - | 4338 | 0.9136 | - |
| Fixed effect | fb | - | 0.09662 | 0.1846 | - |
| Fixed effect | alpha | 1/day | 0.1147 | 15.03 | - |
| Fixed effect | beta | 1/day | 0.004637 | 6.222 | - |
| Fixed effect | C_max_ | Copies/ug | 58450 | 0.08108 | - |
| Fixed effect | T_max_ | Days | 9.364 | 2.635 | - |
| Random effect | foldx | - | 182.8 | 28.37 | 1.00E-10 |
| Random effect | fb | - | 137.2 | 17.88 | 4.718 |
| Random effect | alpha | - | 66.65 | 5.554 | 15.6 |
| Random effect | beta | - | 97.52 | 43.6 | 21.19 |
| Random effect | C_max_ | - | 75.63 | 0.5416 | 5.189 |
| Random effect | T_max_ | - | 26.56 | 0.3354 | 1.00E-10 |
| Residual error | sigma proportional | - | 45.82 | 0.01133 | - |
| Residual error | sigma additive | - | 0.3297 | 0.09618 | - |

C_max_, maximal peripheral CT103A transgene copy number; T_max_, time to maximal expansion; fb, the fraction of persistent CT103A (with contraction rate β) at peak expansion (T_max_); foldx, fold expansion from baseline; RSE, relative standard error of the parameter; Eta shrinkage, shrinkage of empirical Bayes estimates of the parameter, Eta shrinkage for each parameter is calculated by the formula (1 − var(η))/ω 2.

**Table S2: Impact of covariates on the Bayesian posthoc PK parameters from the PopPK base model**

|  | Eta Cmax | Eta Tmax | Eta foldx | Eta fb | Eta α | Eta β | N |
| --- | --- | --- | --- | --- | --- | --- | --- |
| Weight | 0.2514 | 0.4802 | 0.8385 | 0.7937 | 0.4902 | 0.239 | 18 |
| Age | 0.6362 | 0.296 | 0.4032 | 0.9365 | 0.5431 | 0.5501 | 18 |
| Other CART | 0.6693 | 0.4961 | 0.6735 | 0.732 | 0.2786 | 0.3799 | 18 |
| Autotransplant | 0.1026 | 0.5582 | 0.6961 | 0.4314 | 0.0972 | 0.8722 | 18 |
| Extramedullary | **0.0258*** | 0.3472 | 0.376 | 0.229 | 0.0869 | 0.522 | 18 |
| CRS | 0.0539 | 0.065 | 0.1165 | 0.8912 | 0.6288 | 0.9311 | 18 |
| Dose | 0.3748 | 0.2865 | 0.6832 | 0.7444 | 0.161 | 0.7484 | 18 |
| Gender | 0.1796 | 0.7058 | 0.9475 | 0.6475 | 0.2288 | 0.1957 | 18 |

The values above indicate *p*-values; *, *p* < 0.05.

**Table S3: F1 / F2 model PK parameters**

| Type | Parameter | Units | Estimate | RSE (%) | Eta Shrinkage |
| --- | --- | --- | --- | --- | --- |
| Fixed effect | foldx | - | 4111 | 0.4359 | - |
| Fixed effect | fb | - | 0.1304 | 0.05227 | - |
| Fixed effect | alpha | 1/day | 0.0946 | 0.257 | - |
| Fixed effect | beta | 1/day | 0.004866 | 0.05693 | - |
| Fixed effect | C_max_ | Copies/ug | 60330 | 0.3901 | - |
| Fixed effect | T_max_ | Days | 8.354 | 0.04653 | - |
| Fixed effect | F1 | - | 0.9663 | 0.05703 | - |
| Fixed effect | F2 | - | 0.9435 | 0.04658 | - |
| Random effect | foldx | - | 233.2 | 56.12 | 1.00E-10 |
| Random effect | fb | - | 149.6 | 0.2757 | 27.82 |
| Random effect | alpha | - | 96.66 | 0.9043 | 6.186 |
| Random effect | beta | - | 98.67 | 43.96 | 27.49 |
| Random effect | C_max_ | - | 79.5 | 0.1976 | 4.71 |
| Random effect | T_max_ | - | 29.09 | 0.1463 | 13.09 |
| Residual error | sigma proportional | - | 46.93 | 0.000838 | - |
| Residual error | sigma additive | - | 0.332 | 71.49 | - |

F1 and F2 indicate effects of tocilizumab and corticosteroids on the expansion rate respectively; C_max_, maximal peripheral CT103A transgene copy number; T_max_, time to maximal expansion; fb, the fraction of persistent CT103A (with contraction rate β) at peak expansion (T_max_); foldx, fold expansion from baseline; RSE, relative standard error of the parameter; Eta shrinkage, shrinkage of empirical Bayes estimates of the parameter, Eta shrinkage for each parameter is calculated by the formula (1 − var(η))/ω 2.

**Table S4: Summary of modified model individual parameters**

|  | Patient ID | alpha | beta | fb | foldx | Cmax (copies/ug DNA) | Tmax (Days) |
| --- | --- | --- | --- | --- | --- | --- | --- |
| PD group | 01-002 | 0.08349 | 0.024808 | 0.18422 | 1300200 | 75321 | 7.4902 |
|  | 01-013 | 0.2158 | 0.011787 | 0.011561 | 4042.3 | 47856 | 9.4585 |
|  | 01-016 | 0.13753 | 0.006426 | 0.058159 | 13419 | 42779 | 7.8977 |
|  | 01-023 | 0.19512 | 0.003287 | 0.1527 | 427.8 | 11883 | 10.508 |
|  | **Mean** | **0.158** | **0.012** | **0.102** | **329522** | **44459** | **8.84** |
| CR / PR group | 01-001 | 0.037775 | 0.0023 | 0.46431 | 8695.3 | 78785 | 9.2347 |
|  | 01-004 | 0.21422 | 0.003515 | 0.011212 | 50949 | 58650 | 8.2479 |
|  | 01-005 | 0.17464 | 0.003808 | 0.06124 | 25549 | 16822 | 5.8123 |
|  | 01-007 | 0.098274 | 0.004322 | 0.16301 | 6544.7 | 56440 | 8.1464 |
|  | 01-009 | 0.080214 | 0.005884 | 0.76928 | 20109 | 111490 | 8.1006 |
|  | 01-010 | 0.060071 | 0.0029 | 0.108 | 1958.4 | 97839 | 13.523 |
|  | 01-011 | 0.045048 | 0.022642 | 0.056716 | 409100 | 151990 | 6.7294 |
|  | 01-012 | 0.096291 | 0.006101 | 0.12849 | 3268.7 | 58514 | 15.658 |
|  | 01-015 | 0.15721 | 0.019674 | 0.49569 | 42.968 | 39061 | 18.117 |
|  | 01-019 | 0.082374 | 0.001477 | 0.044406 | 8412.3 | 20090 | 7.2139 |
|  | 01-020 | 0.10901 | 0.00435 | 0.12364 | 4514.5 | 82710 | 7.9922 |
|  | 01-021 | 0.1543 | 0.017723 | 1.5709 | 1828.1 | 92084 | 8.8335 |
|  | 01-022 | 0.11744 | 0.005633 | 1.2445 | 7685.6 | 65025 | 8.3055 |
|  | 01-024 | 0.30512 | 0.004286 | 0.11886 | 207670 | 122710 | 6.5474 |
|  | **Mean** | **0.123** | **0.007** | **0.383** | **54023** | **75157** | **9.46** |

**SUPPLEMENTAL FIGURES**

**Figure S1 Good correlation between ddPCR-based quantification of CT103A transgene copy number and flow cytomatry-based CT103A percentage in lymphocytes.**


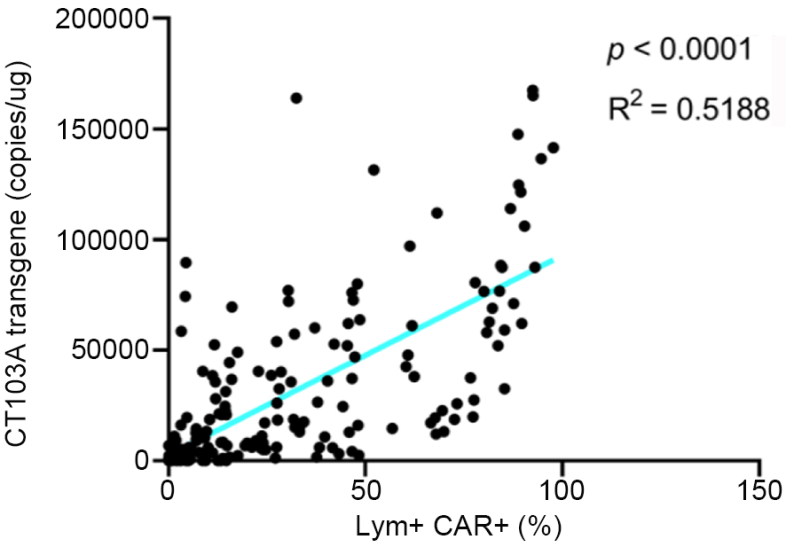


**Figure S2 Diagnostics for base model.** (A) Individual predicted concentration versus observed concentration (left). Population predicted concentration versus observed concentration (right). The red lines represent regression line. (B) Conditional weighted residuals versus time (left) and population predicted concentration (right). The red lines and blue dash lines represent the position where conditional weighted residual equal 0 and ± 4, respectively.


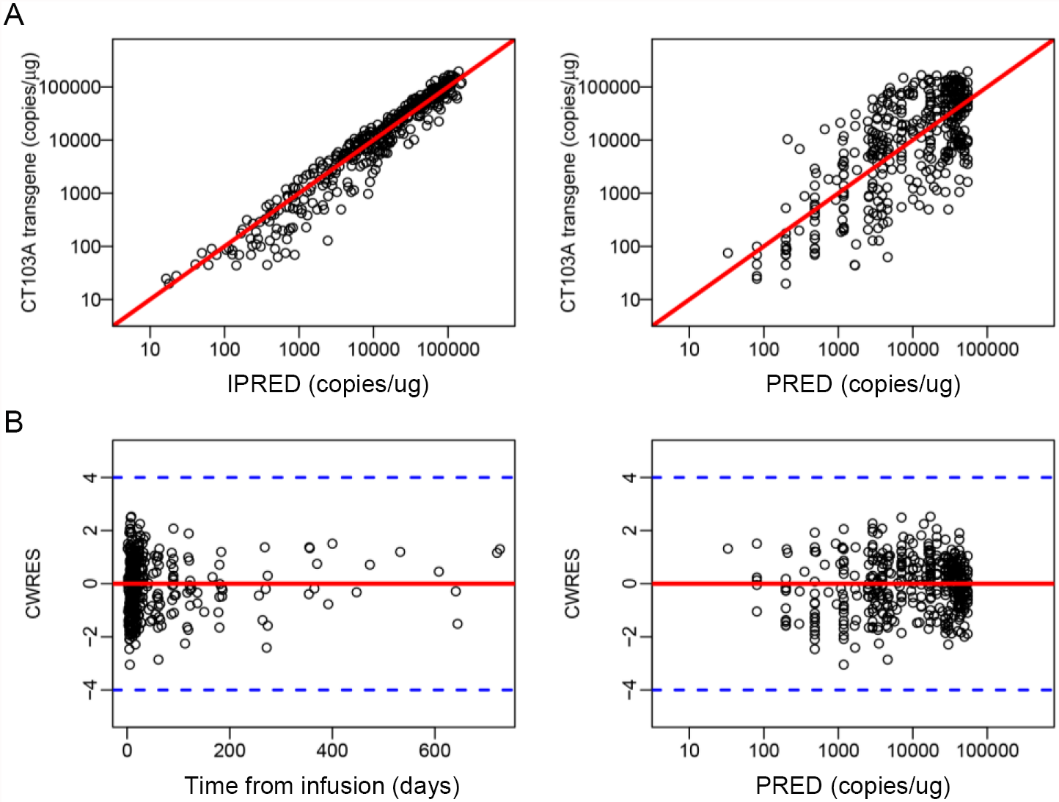


**Figure S3 Impacts of categorical covariates on Bayesian post hoc random effects estimates and AUC_0-28_.**


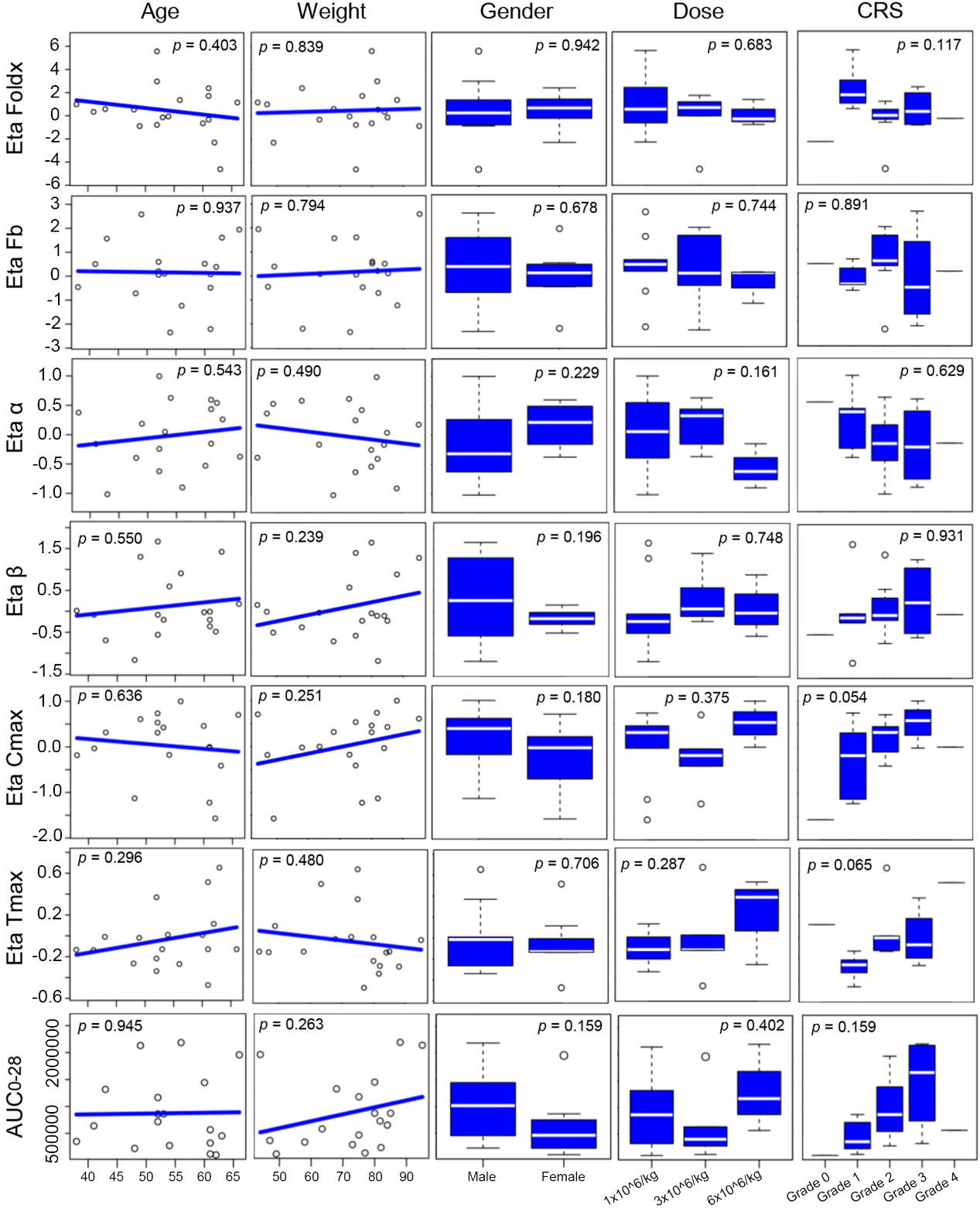

Supplement: Supplementary file 1 [file DataSheet1.docx]
